# Human Serum Albumin Increases the Stability of Green Tea Catechins in Aqueous Physiological Conditions

**DOI:** 10.1371/journal.pone.0134690

**Published:** 2015-07-31

**Authors:** Angelo Zinellu, Salvatore Sotgia, Bastianina Scanu, Mauro Forteschi, Roberta Giordo, Annalisa Cossu, Anna Maria Posadino, Ciriaco Carru, Gianfranco Pintus

**Affiliations:** 1 Department of Biomedical Sciences, University of Sassari, Sassari, Italy; 2 Laboratory of Cell Signaling and Redox Biology, University of Sassari, Sassari, Italy; 3 Quality Control Unit, Hospital University of Sassari (AOU), Sassari, Italy; University of Quebect at Trois-Rivieres, CANADA

## Abstract

Epicatechin (EC), epigallocatechin (EGC), epicatechingallate (ECG) and epigallocatechingallate (EGCG) are antioxidants present in the green tea, a widely used beverage whose health benefits are largely recognized. Nevertheless, major physicochemical limitations, such as the high instability of catechins, pose important questions concerning their potential pharmacological use. Recent studies indicate that binding of catechins with plasmatic proteins may modulate their plasma concentration, tissue delivery and biological activity. After 5 minutes of incubation with HSA both ECG and EGCG were fully bound to HSA, while after 48h incubation only 41% of EC and 70% of EGC resulted linked. HSA had a strong stabilizing effect on all catechins, which could be found in solution between 29 and 85% even after 48h of incubation. In the absence of HSA, EGC and EGCG disappeared in less than 24h, while ECG and EC were found after 48h at 5 and 50%, respectively. The stabilizing effect of HSA toward EGCG, obtained in aqueous physiological conditions, resulted stronger in comparison to cysteine and HCl, previously reported to stabilize this polyphenol. Because of the multitude of contradictory data concerning *in vivo* and *in vitro* antioxidant-based experimentations, we believe our work may shed some light on this debated field of research.

## Introduction

Dietary intake of naturally occurring antioxidants from plant-derived beverage such as green tea has been associated with a reduced incidence of risk factors for a number of chronic and degenerative diseases including cancer and cardiovascular diseases[1–3]. Among the different antioxidants present in green tea, the polyphenolic compounds epicatechin (EC), epigallocatechin (EGC), epicatechingallate (ECG) and epigallocatechingallate (EGCG) are thought to be the major responsible of the health benefits attributed to this widely used beverage [4]. Biological functions of catechins appear strictly related to the number of hydroxyl groups on the B-ring and the presence or absence of a galloyl group. For instance, catechins with a pyrogallol-type structure on the B-ring, such as EGC and EGCG, have strong antioxidant activities [5], whereas catechins with galloyl moiety, such as ECG and EGCG, are more biologically active than their homologues lacking the galloyl moiety [6,7].

Although the health benefits of catechins are largely recognized, major physicochemical limitations, correlated to their structure, pose important questions concerning their biological activities and related dosage. For instance, catechins are highly unstable in solution and degrade through oxidative and epimerization processes in function of the pH and temperature [8], showing to be limited stable at pH below 4 and highly unstable at pH above 6 [9,10]. Nonetheless, pharmacokinetic studies in human have shown that is common to found traces of catechins in plasma and urine even 24 h later of their ingestion, indicating therefore that some sort of stabilization process may occur *in vivo* [11,12]. In this context, it has been reported that, similarly to others compound, dietary flavonoids interact with blood proteins including albumins, which appear involved in their transport to different body's organs and tissues [13–16]. For instance, Human Serum Albumin (HSA) has several drug-binding sites including eleven for medium- and long-chain fatty acids, and two for aromatic and heterocyclic compounds [17,18], which might be involved in catechins stabilization as previously reported for EGCG and resveratrol [19,20]. Binding of catechins to plasma protein, may also account for the different biologically active dose between *in vitro* and *in vivo* experiments reported in literature [21]. Indeed concentrations that appear responsible for the *in vitro* biological effects of catechins are remarkably higher as compared with the ones found in the plasma *in vivo* [21]. Hence, as reported for the polyphenol resveratrol [22,23], this bind may serves as modulator of catechins plasma concentration, tissue delivery and biological activity.

Consonant with the importance of the above-mentioned interaction, we have recently described a study for the binding analysis of EC, EGC, ECG and EGCG with HSA, which resulted in the determination of the catechins binding constant toward this plasmatic protein [24]. However, considering the effect of the above-reported interaction on catechins stability, apart from EGCG [19], no data have been so far reported on the effect of HSA on the others main green tea catechins EC, EGC and ECG. Hence, this work have been undertaken to investigate whether the binding with HSA may affect catechins stability in aqueous physiological conditions in comparison to their free form.

## Results

### The binding of gallate catechins influences peak area and migration time of HSA

**Fig 1:**
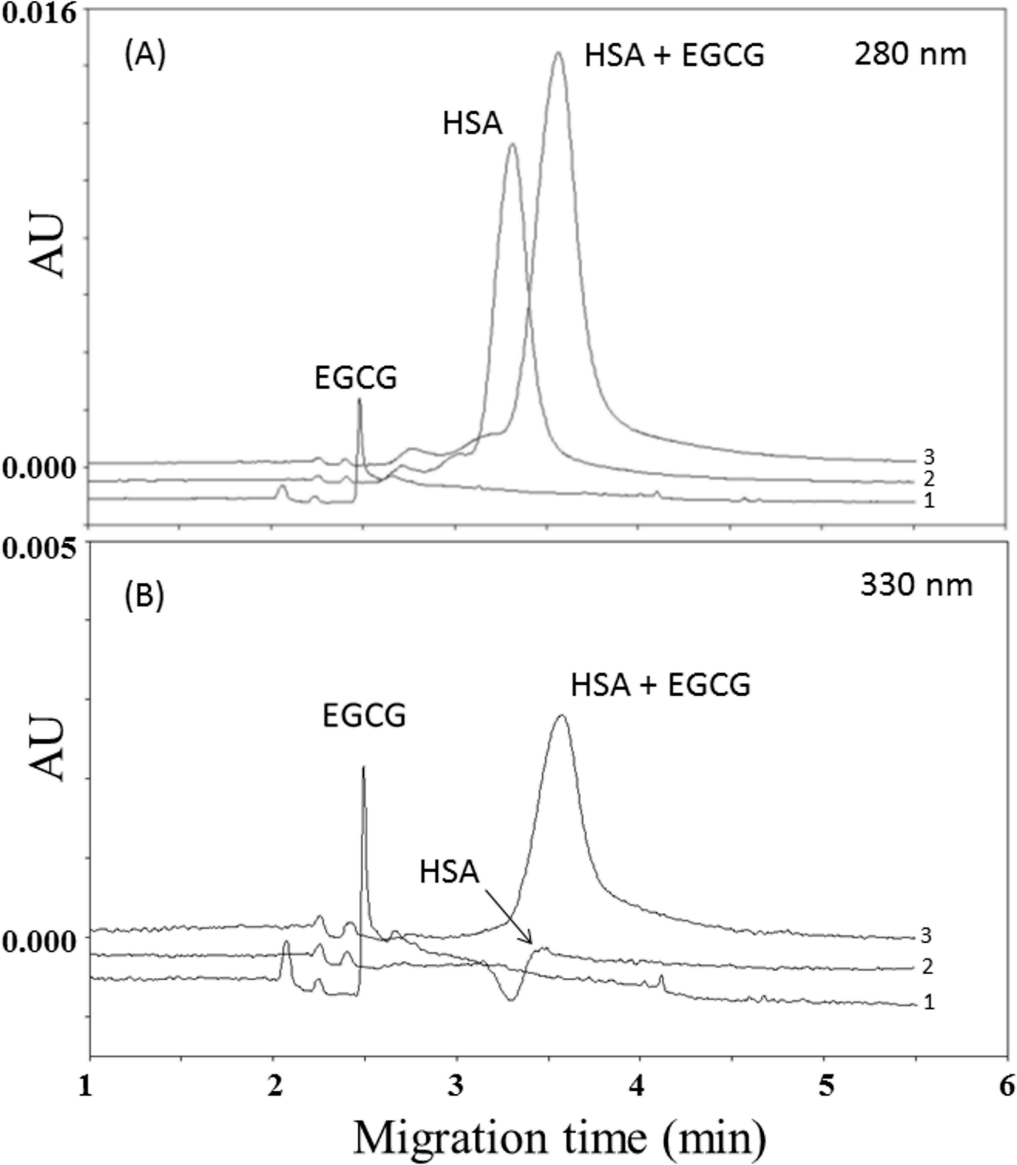
Electropherograms of (1) 0.5 mmol/L EGCG, (2) 0.750 mmol/L human serum albumin (HSA) and (3) mix of 0.5 mmol/L EGCG and 0.750 mmol/L human serum albumin (HSA) monitored at 280 nm (panel A) and 330 nm (panel B).

Under non-oxidative conditions, polyphenols form reversible non-covalent complexes with HSA, either through hydrogen bonding or hydrophobic interactions [13,14]. To gain insight concerning potential interactions between HSA and the main green tea catechins, the protein (1.500 mmol/L in PBS) was incubated with EC, EGC, ECG and EGCG (1 mmol/L in PBS) for 48 h at 25°C. Then aliquots of 200 μl of the above solution were taken at different time points (0, 5 minutes, 3 h, 8 h, 24 h and 48 h) and directly injected on capillary electrophoresis UV detection in 10 mmol/L phosphate buffer at pH 7.4. As reported in Fig 1A, analysis of the mix (EGCG-HSA) performed at 280 nm (wavelength of maximum absorbance of proteins) show the complete disappearance of the EGCG peak (electropherogram 3), which was instead present when the EGCG was singularly injected (electropherogram 1). The same figure indicates that the HSA peak had both a shift and an increase in peak area (about 22% of the initial concentrations of HSA, which was set as 100%) respect to when the protein was singularly injected (electropherogram 2), thus suggesting that all the EGCG present in the mix was linked to albumin. The increased relative electrophoresis mobility (REM) of HSA as compared to HSA-EGCG (from 7.66 ± 0.12 x 10^−3^ cm^2^V^-1^S^-1^ to 7.91 ± 0.15 x 10^−3^ cm^2^V^-1^S^-1^, p<0.05) indicated that HSA has become more anionic probably because of the contribution of the gallate group of EGCG.

The set of analysis performed at 330 nm, which under our experimental conditions was one of the wavelengths of maximum absorbance of catechins, shows the complete disappearance of the EGCG peak in the EGCG-HSA mix (electropherogram 3), which was instead present when the EGCG was injected in the absence of HSA (electropherogram 1) (Fig 1B). The same figure also report an increase of HSA peak amplitude when the EGCG was linked to the protein (electropherogram 3), which was instead absent when the HSA was injected in the absence of EGCG (electropherogram 2). Results similar to those reported in Fig 1A and 1B were found for all the analyzed time-points (not shown). By performing the experiments with ECG, an increase in the HSA peak area (around 19%) was recorded when the protein was linked to ECG, which gave origin to a shift from 7.71±0.09 x 10^-3^cm^2^V^-1^S^-1^ to 7.88±0.12 x 10^−3^ cm^2^V^-1^S^-1^ (p<0.05) of the HSA REM. Similar data were found for all the analyzed time-points (not shown).

### The binding of non-gallate catechins does not influences peak area and migration time of HSA

**Fig 2:**
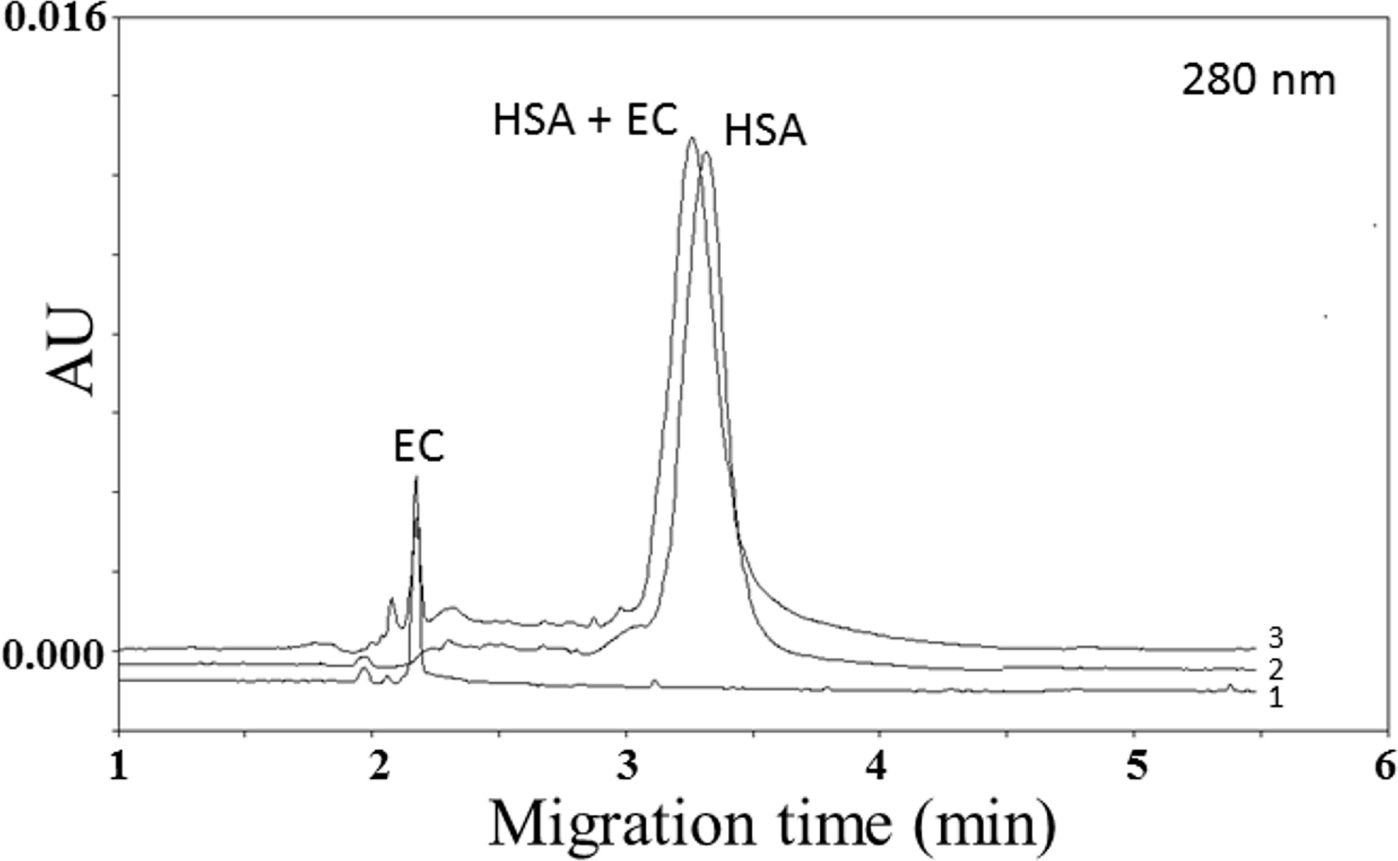
Electropherograms of (1) 0.5 mmol/L EG, (2) 0.750 mmol/L human serum albumin (HSA) and (3) mix of 0.5 mmol/L EGCG and 0.750 mmol/L human serum albumin (HSA) monitored at 280 nm.

**Fig 3:**
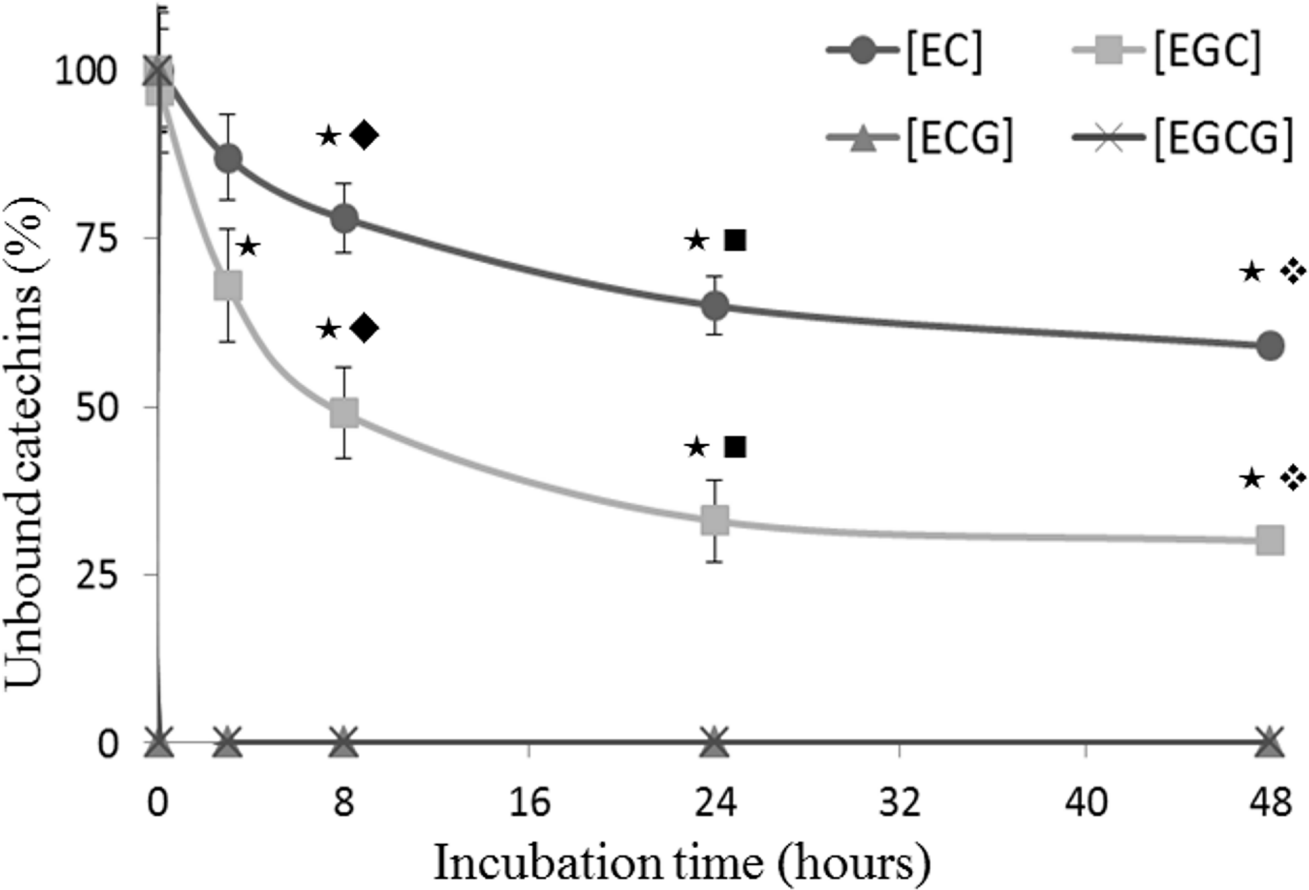
Unbound fraction of EC, EGC, ECG and EGCG assessed during incubation with human serum albumin. 0.750 mmol/L of human serum albumin (HSA) was separately incubated with 0.5 mmol/L of each catechins at 25°C for 48h. During the incubation period, aliquots of 200 μl of the mixture were taken at different time points (0,5 minutes, 3h, 8h, 24h and 48h) and directly injected on capillary electrophoresis to evaluate unbound catechins. The time-points associated this symbol (★) are significantly different from the point at time zero; the time-points associated this symbol (◆) are significantly different from each other; the time-points associated this symbol (■) are significantly different from each other; the time-points associated this symbol (❖) are significantly different from each other.

Fig 2 reports the behavior of non-gallate catechins, which was rather different from the gallate ones. Even after 48 h of incubation, the analysis of the EC-HSA mix revealed a weak interaction, which was indeed unable to produce a significant variation of the EC-HSA REM in comparison to the HSA singularly injected (7.59 ± 0.11 x 10^−3^ cm^2^V^-1^S^-1^ to 7.66 ± 0.12 x 10^−3^ cm^2^V^-1^S^-1^ respectively, p = 0.36). Consistently, determination of the peak area values at 280 nm did not shown significant difference between bound and unbound HSA (+2.8% for HSA-EC *vs* HSA alone, p = 0.79). Similar results were also obtained after 48h-incubation of HSA with EGC (Fig 3). However, in comparison to EC (about 59% of EC was unbound to albumin), after 48 h of incubation the value of EGC unbound to albumin was about 30%, indicating a significant pronounced affinity of the later catechin toward HSA (Fig 3). The same figure reports the 48-h kinetic value of unbound catechins for EC, EGC, ECG and EGCG (% respect to initial concentrations of catechins added set as 100%), which clearly indicates that during the whole time-course EGC was more affine toward HSA as compared to EC. We were unable got a similar kinetic data for EGCG and ECG. Indeed, the amount of unbound gallate catechins was undetectable right after the injection of the first time point (time 0) and so remained during the whole time-course (Fig 3).

### Binding of HSA has a stabilizing effect on catechins

**Fig 4:**
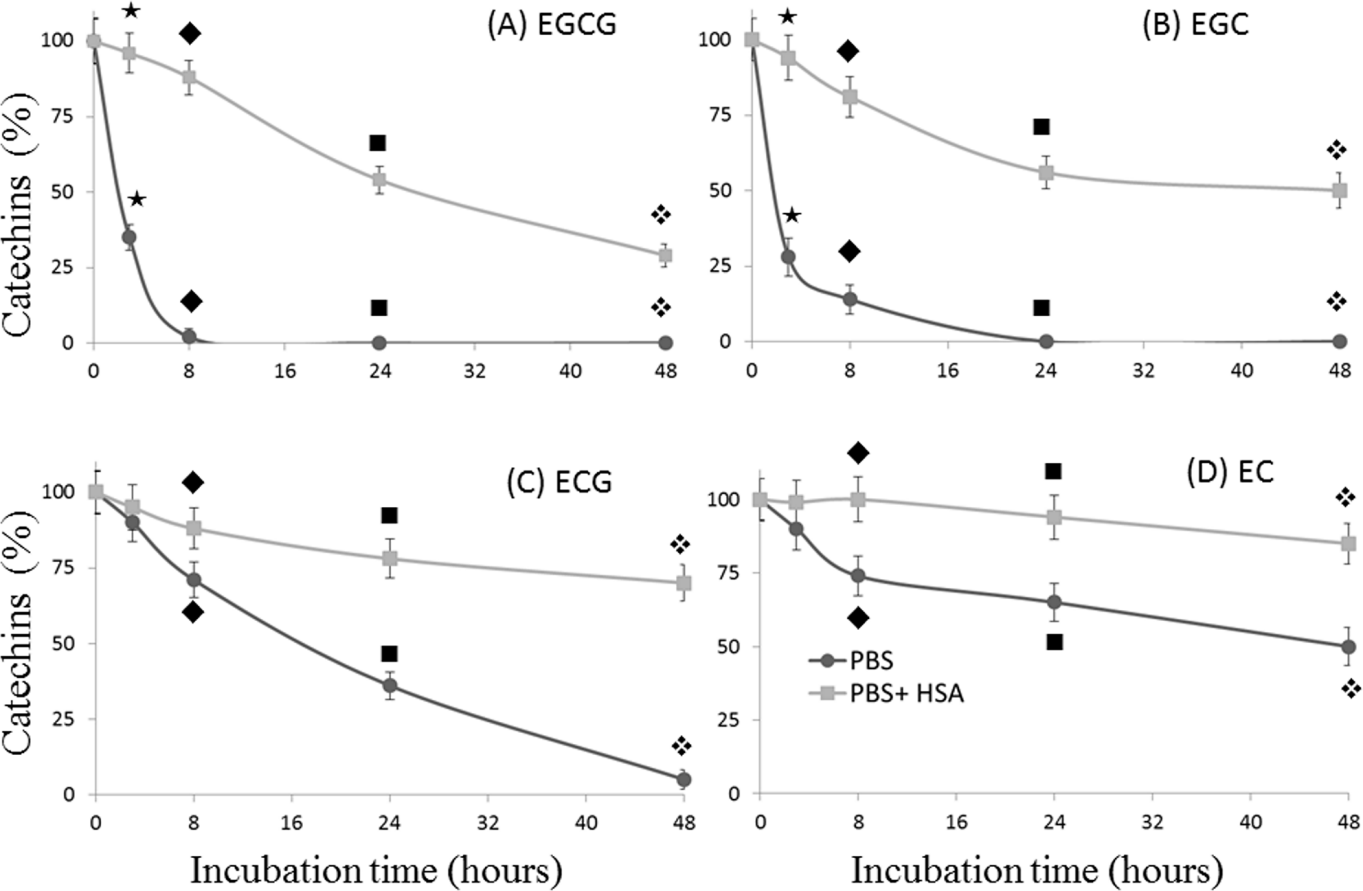
Stabilizing effect of human serum albumin (HSA) on green tea catechins. Each catechin (0.5 mmol/L in PBS) was separately incubated for 48 h at 25°C both in the presence and in the absence of HSA (0.750 mmol/L in PBS). During the incubation period, 200 μL of the mixture has been taken at different time points (0, 3h, 8h, 24 h and 48 h), mixed with 100 μL of acetonitrile and centrifuged to precipitate HSA. The resulting supernatant has been directly injected on capillary electrophoresis UV detection. For each different panel, the time-points associated this symbol (★) are significantly different from each other; the time-points associated this symbol (◆) are significantly different from each other; the time-points associated this symbol (■) are significantly different from each other; the time-points associated this symbol (❖) are significantly different from each other.

**Fig 5:**
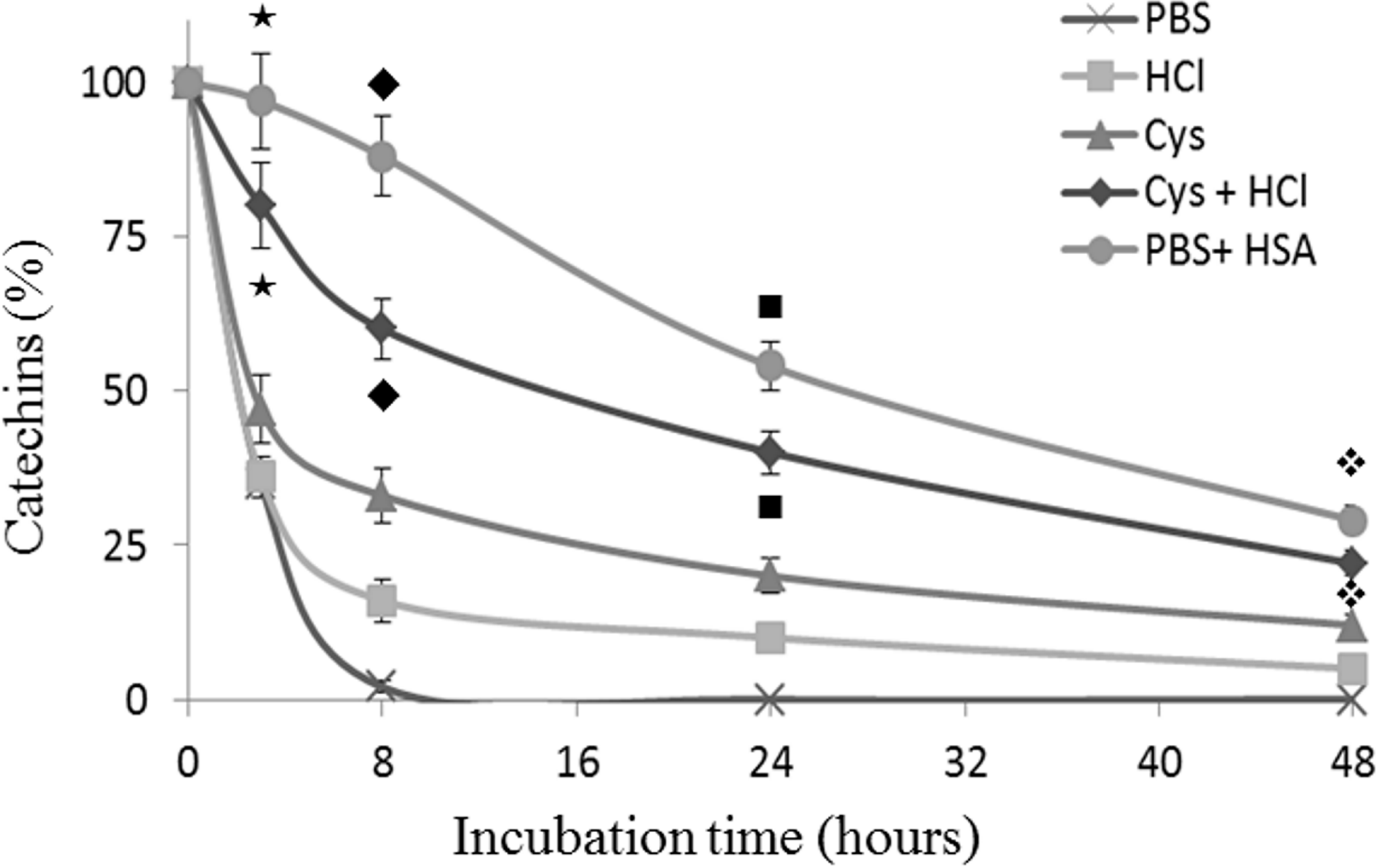
Comparison between the stabilizing effect of HCl, cysteine and human serum albumin on EGCG. 0.5 mmol/L of catechin was separately incubated at 25°C for 48 h with only PBS, 10 mmol/L of cysteine in PBS, 50 mmol/L of HCl, 10 mmol/L of cysteine in 50 mmol/L HCl, or 0.750 mmol/L of Human Serum Albumin (HSA) in PBS. During the incubation period, 200 μL of the mixture has been taken at different time points (8h, 24 h and 48 h), mixed with 100 μL of acetonitrile and centrifuged to precipitate HSA. The resulting supernatant has been directly injected on capillary electrophoresis UV detection to evaluate residual catechin. The time-points associated this symbol (★) are significantly different from each other; the time-points associated this symbol (◆) are significantly different from each other; the time-points associated this symbol (■) are significantly different from each other; the time-points associated this symbol (❖) are significantly different from each other.

Next, we sought to investigate whether the binding of HSA with green tea catechins may affect their stability in aqueous solution. To this end, EC, EGC, ECG and EGCG were incubate with PBS both in the presence and in the absence of HSA and aliquots of 200 μl were analyzed at different time points during a 48 h period. As reported in Fig 4, incubation of catechins in PBS resulted in a significant time-dependent decrease of the analyte concentration. The presence of HSA in solution allows catechins preservation even after 48 h of incubation, which were indeed found in the following percentage respect to the initial concentration set as 100%: 29% of EGCG (vs 0% in PBS) panel A; 50% of EGC (vs 0% in PBS) panel B; 70% ECG (vs 5% in PBS) panel C and 85% EC (vs 50% in PBS) panel D. The stabilizing effect of HSA toward catechins was particularly evident for the less stable catechins, those harboring the pyrogallol-type structure on the B-ring EGCG and EGC, which were found at 88 and 81%, respectively, vs 2 and 14% when solubilized in PBS without HSA (data related to the point at 8 h in Fig 4). In this context, It has been reported that catechins, with particular reference to EGCG, could be stabilized by acidic conditions[25–27], moreover recent observations indicate as the same catechin was stabilized for at least one hour in PBS containing 1 mmol/L cysteine [19]. However, the above-reported experimental data, besides to being run in non-physiological conditions, have been obtained employing a very short incubation times. Hence, we wondered whether HSA may have a better and long-lasting stabilizing effect as compared to both cysteine and acid conditions. To this end, we compared the stabilizing effect of HSA, cysteine and HCl on ECGC during a longer interval time (48 of incubation at room temperature). As reported in Fig 5, at least until 24 h of incubation the presence of HSA in the buffer resulted in the preservation of a significantly higher quantity of catechins as compared to all the others conditions.

## Discussion

It has been suggested that binding of catechins to plasma proteins, including human serum albumin (HSA), may serve as natural in vivo carrier and reservoir modulating thus their bioavailability and eventually their biological activity. In this context, two major binding regions, namely site-I and site-II, have been reported to be responsible for the ability of HSA to bind aromatic and heterocyclic compounds [28,29]. These domains, which are characterized by the presence of a central cavity formed from six amphiphatic helices, have been recently reported to be the same that are responsible for the binding of HSA with catechins [30]. It may be possible that by acting through hydrogen bonding and hydrophobic interactions these polyphenolic compounds form reversible non-covalent complexes with HSA. In fact, interactions among both hydroxyl groups and aromatic rings of catechins with the amino acid residues in the protein chain are highly conceivable [31]. Consonant with our previously data [24], the current findings confirm that catechins harboring a galloyl moiety (ECG and EGCG) have higher binding affinity toward HSA than the catechins lacking it (EC and EGC). Indeed, after 5 minutes of incubation with HSA, both ECG and EGCG were fully bound to protein, while even after 48h incubation only the 41% of EC and 70% of EGC resulted linked to albumin (Figs 1 and 3).

Catechins have poor stability in neutral or alkaline solutions and their instability in several cell culture media has been reported [8–10,32]. In general, catechins are stable in acidic solution, and the neutral or slightly alkaline environment of the body has been suggested to increase their instability [19]. Nevertheless, previous reports indicate that traces of catechins are detectable in blood and urine even 24 h after oral administration in mammals including humans [11,12], and measurements of EGCG levels in human, rat, and mouse blood show a half-life of about 5 h [33]. Thus, a sort of yet unclear stabilization mechanism should be present to maintain these concentrations of catechins in the blood. Recently Bae et al. have suggested that albumin may be responsible for the steadying of EGCG in plasma, at the least for 1 h of incubation period [19]. Our current data, beside confirms these results, demonstrated that the HSA stabilizing effect on EGCG can last until 48h of incubation (Fig 4). We also recorded for the first time a strong stabilization effect of HSA on the other green tea catechins. In particular, after 48h of incubation with HSA, a percentage between 29 and 85% was preserved, while in absence of the protein, EGC and EGCG disappeared in less than 24 hours, and ECG and EC were still detected after 48h at a percentage of 5 and 50%, respectively. Considering that a small fraction of catechins may covalently bind albumin also in our mild conditions [34] we hypothesize that the reported stabilizing effect might be underestimated, since this minor fraction of "HSA-catechins" could precipitate with albumin during the extraction procedure with acetonitrile. Although the fine mechanism involved in the observed stabilization process remains to be fully elucidated, the current data provides direct evidence that HSA is able to stabilize catechins under physiological aerobic condition.

HSA is a non-glycosylated, single-chain polypeptide tightly folded into three domains that are structurally defined by 17 intra-chain disulfide bonds formed between 34 cysteine residues. The HSA cysteine residue at Cys 34, which does not participate in the formation of intra-chain disulfide bonds, by actively interacting with reactive oxygen and nitrogen species, has been proposed to have an important antioxidant role [35]. Recent observations suggest that the–SH group of albumin, acting as an effective scavenger of reactive species, may protect catechins from degradation [19]. The authors justified this hypothesis by the observation that both cysteine and GSH do have a similar protective ability toward catechins whereas other non-thiolate amino acids do not! Our current data indicated that the cysteine (10 mmol/L) had a less protection than the HSA (0.750 mmol/L), a result even accentuated by the fact that the employed HSA contained 0.24 mol-SH/mol protein, therefore 0.750 mmol/L of HSA provided a real quantity of–SH of 0.18 mmol/L. Hence after 48 hours of incubation, a concentration 50 times smaller of-SH protein had more than double of protective effect if compared with the one elicited by the cysteine. Interestingly, the scavenger ability of albumin–SH groups seems to participate in the protection of catechins from oxidative processes, especially in the first phases of incubation as described by Bae et al. [19], although our findings suggest that other factors may be involved for longer incubation times.

The interaction of plasmatic proteins with natural polyphenols appears to modulate their plasma concentration and tissue delivery [22,36–39]. HSA is the most abundant protein in the human blood plasma, therefore establishing all the parameters, which regulate it’s interaction with natural antioxidant, and eventually whether it may or may not influence their biological activities, remains a crucial aspect to be investigate. Here we reported for the first time that HSA is able to modulate catechins stability in aqueous solution, being able to preserve these compounds in a significantly large amount even after long incubation times. Because of the multitude of contradictory data related to the pharmacological employment of natural antioxidants, we believe our work may shed some light on this debated research field.

## Material and Methods

### Chemicals

EGC, EC, ECG, EGCG, HSA, Na_2_HPO_4_•2H_2_0, NaH_2_PO_4_ •H_2_0, phosphoric acid, hydrochloric acid, boric acid, sodium hydroxide, acetonitrile, were obtained from Sigma (St. Louis, USA). 0.45 μm membrane filters (used to filter all buffer solution before CE analysis) were purchased from Millipore (Bedford, USA).

### Standards preparation

Individual standard catechins EGC, EC, ECG, EGCG were dissolved in water at 1 mmol/L final concentration. HSA stock solution (1.500 mmol/L) was prepared by dissolving the powder of definite weight in deionized water. All the stock solutions were stored at -80°C until used.

### Analysis of HSA and catechins interaction1

100 μL of catechins (1 mmol/L in PBS) was mixed with 100 μL of HSA (1.500 mmol/L in PBS) or with 100 μL of PBS as control. Then the mixture was incubated at 25°C for 48 h and interaction between catechins and HSA was checked by direct injection of 100 μl on capillary electrophoresis at the indicated time-points (0, 5 minutes, 3 h, 8 h, 24 h and 48 h). A MDQ capillary electrophoresis system equipped with a diode array detector was used (Beckman instruments, CA, USA). The system was fitted with a 30 kV power supply with a current limit of 300 μA. Analysis was performed in an uncoated fused-silica capillary, 75 μm I.D. and 40 cm length (30 cm to the detection window), injecting 45 nl of sample (3.45 kPa x 5 seconds). Separation was carried out in a 10 mmol/L sodium phosphate pH 7.4, 37°C and 15 kV at normal polarity. After each run, capillary was rinsed with, 1 min of 1 mol/L NaOH, 1 min of 0.5 mol/L NaOH, and equilibrated with run buffer for 1 min. In the adopted electrophoretic conditions, the absorbance spectrum of catechins gave three absorbance peaks for the gallate catechins (214, 275 and 330 nm) and two for the non-gallate counterparts (210 and 274 nm). On the base of the analysis performed, the reading conditions that gave the best result where: Absorption at 214 nm (single catechins), 280 nm (HSA-catechins, with reference to HSA) and 330 nm (HSA-catechins, with reference to catechins). For each analyte, the variation of peak area was expressed as percentage respect to the peak area of the reference compound set as 100%.

### Determination of catechins stability

To evaluate catechins stability 100 μL of catechins (1 mmol/L in PBS) was mixed with 100 μL of HSA (1.500 μmol/L in PBS) or with 100 μL of PBS as control. The mixture was incubated at 25°C for 48 h, during the incubation time aliquot of 100 μl were taken the indicated time-points (0, 5 minutes, 3h, 8h, 24 h and 48 h), mixed with 100 μL of acetonitrile and centrifuged 2000g x 5 min to precipitate albumin. The resulting supernatant was directly injected on capillary electrophoresis UV detection. Analysis was performed in an uncoated fused-silica capillary, 75 μm I.D. and 40 cm length (30 cm to the detection window), injecting 45 nl of sample (3.45 kPa x 5 seconds). Separation was carried out in a 200 mmol/L sodium borate pH 8.4, 37°C and 15 kV at normal polarity for 6 minutes. Migration of analytes was monitored by absorption at 214 nm (wavelength of maximum absorbance of catechins in PBS). After each run, capillary was rinsed with, 1 min of 1 mol/L NaOH, 1 min of 0.5 mol/L NaOH, and equilibrated with run buffer for 1 min. Data were expressed as percentage respect to the initial amount of catechins added set as 100%.

### Statistical analysis

Data are expressed as means ± S.D. of four or five different experiments. Kinetic interaction between catechins and HSA was evaluated by One-way analysis of variance (ANOVA) followed by a post-hoc Bonferroni Comparison Test to detect differences of means among the control (time = 0) and the different time-points with significance defined as P < 0.05. The statistical differences among catechins incubated in PBS and HSA were assessed using the Student’s t-test with significance defined as P < 0.05. Statistical analysis were performed using MedCalc for Windows, Version 12.5 64 bit (MedCalc, Software, Ostend, Belgium) and SPSS for Windows, Version 14.0 32 bit (IBM Corporation, Armonk, NY, USA).
